# Rapid and Robust PCR-Based All-Recombinant Cloning Methodology

**DOI:** 10.1371/journal.pone.0152106

**Published:** 2016-03-23

**Authors:** Abhishek Anil Dubey, Manika Indrajit Singh, Vikas Jain

**Affiliations:** Microbiology and Molecular Biology Laboratory, Department of Biological Sciences, Indian Institute of Science Education and Research (IISER), Bhopal, India; New Mexico State University, UNITED STATES

## Abstract

We report here a PCR-based cloning methodology that requires no post-PCR modifications such as restriction digestion and phosphorylation of the amplified DNA. The advantage of the present method is that it yields only recombinant clones thus eliminating the need for screening. Two DNA amplification reactions by PCR are performed wherein the first reaction amplifies the gene of interest from a source template, and the second reaction fuses it with the designed expression vector fragments. These vector fragments carry the essential elements that are required for the fusion product selection. The entire process can be completed in less than 8 hours. Furthermore, ligation of the amplified DNA by a DNA ligase is not required before transformation, although the procedure yields more number of colonies upon transformation if ligation is carried out. As a proof-of-concept, we show the cloning and expression of *GFP*, *adh*, and *rho* genes. Using GFP production as an example, we further demonstrate that the *E*. *coli* T7 express strain can directly be used in our methodology for the protein expression immediately after PCR. The expressed protein is without or with 6xHistidine tag at either terminus, depending upon the chosen vector fragments. We believe that our method will find tremendous use in molecular and structural biology.

## Introduction

Polymerase chain reaction (PCR) has revolutionized the recombinant DNA technology. Generally, to construct a recombinant molecule, the desired DNA fragment is prepared in large quantity using PCR and the amplicon (the PCR-amplified DNA) thus obtained is cloned in the vector of choice. This cloning is carried out by following any of the several available methods [1–10]. Furthermore, in order to obtain a protein product, the cloning of the amplicon is carried out in an expression vector such as pET vectors (Novagen, Madison, WI, USA). Construction of the clones that are used for the production of proteins usually require careful designing of primers, selection of restriction enzymes, and digestion of both the vector and the amplicon followed by ligation of the two. Thus the involvement of several steps makes it difficult to rapidly achieve the desired cloning with high efficiency. Several methodologies such as the ligation-independent cloning and the recombination-based Gateway cloning are now available that allow bypassing of one or more steps in the molecular cloning process [11,12]. For example, ligation independent cloning avoids the use of phosphorylation of the PCR-amplified DNA product, digestion by the restriction enzymes, and the ligation using a DNA ligase [11]. Similarly, gateway cloning allows the subcloning of any gene from the gateway vector to other vectors without using restriction enzymes [12].

Cloning of the desired gene directly in an expression vector for protein production saves time. We earlier reported the engineering of three expression vectors that allowed the cloning of a blunt-end PCR product directly into an expression vector and provided 6x-His tag at either terminus [13]. Additionally, one of the quick-series vectors allowed the expression of the recombinant protein with no tag [13]. These vectors were designed considering a general practice in the protein expression and purification experiments, i.e. fusing the protein of interest with an affinity tag for rapid purification [14]. Hexa-histidine tag, which is one of the most widely used affinity tags, allows one-step purification of the recombinant proteins even in the presence of denaturants such as urea and guanidine hydrochloride [15]. In this paper, we report an all-PCR based cloning methodology that is extremely rapid involving only two PCR reactions, and does not require any digestion or modification of the PCR product. Using our methodology, obtaining a non-recombinant clone is extremely unlikely due to the following reason. Here we have separated the two required features of the vector, i.e. origin of DNA replication (*ori*) and antibiotic-resistance marker, on two individual DNA fragments. These elements are required in *cis* for a plasmid to survive in the bacterial cell. The two fragments can be joined after an overlapping PCR only when the gene of interest is present in the middle and has vector-complementary ends. This thus completely eliminates the possibility of getting non-recombinant clones. After insertion of amplicon, the recombinant vector is amplified in subsequent PCR cycles using phosphorylated primers. The final product requires no digestion, phosphorylation or ligation, and is directly transformed in *E*. *coli* XL1-blue strain. To demonstrate the efficiency and reliability of our methodology, we have performed the subcloning of *GFP* gene from a pET21b vector, and the cloning of *adh* and *rho* genes from *Mycobacterium smegmatis* and *Escherichia coli*, respectively.

## Materials and Methods

### Bacterial strains, media, and growth conditions

*E*. *coli* strain XL1-Blue (Stratagene) was used for cloning, whereas BL21(DE3) (Lucigen) and T7 express (New England Biolabs) strains were used for the protein expression. Bacteria were grown in LB broth at 37°C in the presence of 100 μg/ml ampicillin (Amp) with constant shaking at 200 rpm in all experiments. Protein expression was induced with IPTG to a final concentration of 1 mM. The standard microbiological and molecular biology methodologies and protocols were adopted in all experiments [16].

### Reagents

Restriction enzymes, DNA modifying enzymes, High fidelity DNA polymerases Phusion and Q5 were procured from either NEB or Fermentas, as specified, and were used as per manufacturer’s instructions. All other reagents were obtained from Sigma Aldrich and were of highest quality available. The primers used in PCR and site directed mutagenesis (SDM) were purchased from Macrogen (South Korea).

### Site directed mutagenesis

SDM was performed using single primer approach using ori-Amp-SmaI primer essentially as described elsewhere [17]. The mutation was confirmed by restriction digestion of the isolated plasmid.

### Digestion of vector by SmaI

20 μg of each plasmid (pRS-NHS, pRS-CHS and pRS-NOHS) was digested with 40 units of SmaI enzyme in presence of 1x buffer supplied by the manufacturer. The reaction was incubated for 2 hours at 25°C. The two DNA fragments generated after the digestion were separated on agarose gel and purified using a DNA gel extraction kit (Qiagen), and quantified.

### PCR and cloning

#### Amplification of genes

PCR was performed to amplify respective genes with different primer pairs. Each primer pair (Table 1) either provided overhang complementary to pMSQSNHS, pMSQSCHS, or pMSQSNOHS. All the three genes were amplified with three different primer pairs considering cloning of the gene in all three vectors. The PCR product was gel extracted and quantified before the final overlapping PCR reaction. The *GFP* was amplified from pET21b vector, whereas *rho* and *adh* genes were amplified from *E*. *coli* BL21(DE3) and *M*. *smegmatis* mc^2^155 genomic DNA, respectively using the primer sequence as shown in the Table 1.

**Table 1:** List of primers used in this study. The underlined sequence is complementary to the plasmid sequence and is required for overlapping PCR.

Primer	Sequence (5’-3’)
^1^ori-Amp-SmaI	CCACTGGCAGCAG***CCCGGG***GTAACAGGATTAGCAGAGC
^2^Ori-For	GGGCTGCTGCCAGTGGCGATAAGTCG
^2^Amp-For	GGGGTAACAGGATTAGCAGAGCGAGG
rho-CHS-for	GTTTAACTTTAAGAAGGAGATATCCCATGAATCTTACCGAATTAAAGAATACG
rho-CHS-rev	CTCAGTGGTGGTGGTGGTGGTGCCCTGAGCGTTTCATCATTTCGAAGAAATCG
rho-NHS-for	GCCTGGTGCCACGCGGCAGCCCCATGAATCTTACCGAATTAAAGAATACG
rho-NHS-rev	GCTTTGTTAGCAGCCGGATCTCACCCTGAGCGTTTCATCATTTCGAAGAAATCG
adh-CHS-for	GTTTAACTTTAAGAAGGAGATATCCCATGAAAACCAAAGCCGCCGTGAGC
adh-CHS-rev	CTCAGTGGTGGTGGTGGTGGTGCCCGGCTTCCGGATCGTGGATGATGACG
adh-NHS-for	GCCTGGTGCCGCGCGGCAGCCCCATGAAAACCAAAGCCGCCGTGAGC
adh-NHS-rev	GCTTTGTTAGCAGCCGGATCTCACCCGGCTTCCGGATCGTGGATGATGACG
GFP-CHS-for	GTTTAACTTTAAGAAGGAGATATCCCATGAGCAAGGGCGAGGAGCTGTTCACCG
GFP-CHS-rev	CTCAGTGGTGGTGGTGGTGGTGCCCCTTGTACAGCTCGTCCATGCCGAGAGTG
GFP-NHS-for	GCCTGGTGCCACGCGGCAGCCCCATGAGCAAGGGCGAGGAGCTGTTCACCG
GFP-NHS-rev	GCTTTGTTAGCAGCCGGATCTCACCCCTTGTACAGCTCGTCCATGCCGAGAGTG
PETFOR	ATCGAGATCTCGATCCCGCGAAATTAATACG
T7rev	GCTAGTTATTGCTCAGCGG

^1^ SmaI site is boldfaced and italicized.

^2^ These primers were designed with and without 5’phosphate.

#### Formation of recombinant DNA

The PCR reaction was performed using Q5 high fidelity polymerase (NEB). The reaction mixture contained 0.5 ng of vector fragments, 10 ng of gene amplicon, 200 nM of each phosphorylated primer (OriFor and AmpFor) and 200 μM of dNTPs mixture in 1x reaction buffer (Q5 buffer, NEB). The primers were annealed to the template at 64°C for cloning genes in pMSQSNHS, 62°C for cloning in pMSQSCHS, and 59°C for cloning in pMSQSNOHS. The PCR product was either directly transformed into bacteria or was subjected to PCR clean up (Qiagen) before transformation. The purified PCR product was further ligated and transformed into bacterial cells. The *rho* and *adh* genes containing recombinant molecules were transformed in XL-1 blue strain of *E*. *coli* while the PCR reaction mix for *GFP* gene was transformed in *E*. *coli* T7 express cells; the latter was plated on LB-agar containing 100 μg/ml Amplicillin and 1 mM IPTG, to monitor the percentage positive cells by directly visualizing GFP expression in colonies.

### Screening of positive clones and induction of protein

Although recombinant clones for *GFP* were screened by visualizing the green coloured colonies on the LB-Amp-IPTG plate, for *adh* and *rho*-positive clones were confirmed by colony PCR (cPCR). The reaction conditions were same as reported previously [13]. Colony PCR was performed using DreamTaq Green Master Mix (Fermentas) and the pETFor and T7rev primers were used to amplify the gene of interest (Table 1). The plasmids were extracted from the positive clones and transformed in *E*. *coli* BL21(DE3) cells to assess protein expression. The bacteria were grown in LB broth until the optical density at 600 nm reached ~0.6; IPTG to a final concentration of 1 mM was then added. Cells were subsequently harvested by centrifugation at high speed and lysed in lysis buffer containing 40 mM TrisCl pH 8.0, 200 mM NaCl, and 8 M urea. The lysate was centrifuged again at high speed and the proteins were separated on a 12% SDS-PAGE gel that was stained with Coomassie dye and imaged.

## Results

### Generation of pRS vector fragments separating the origin of replication (*ori*) and the ampicillin antibiotic resistance marker (*bla*)

**Fig 1:**
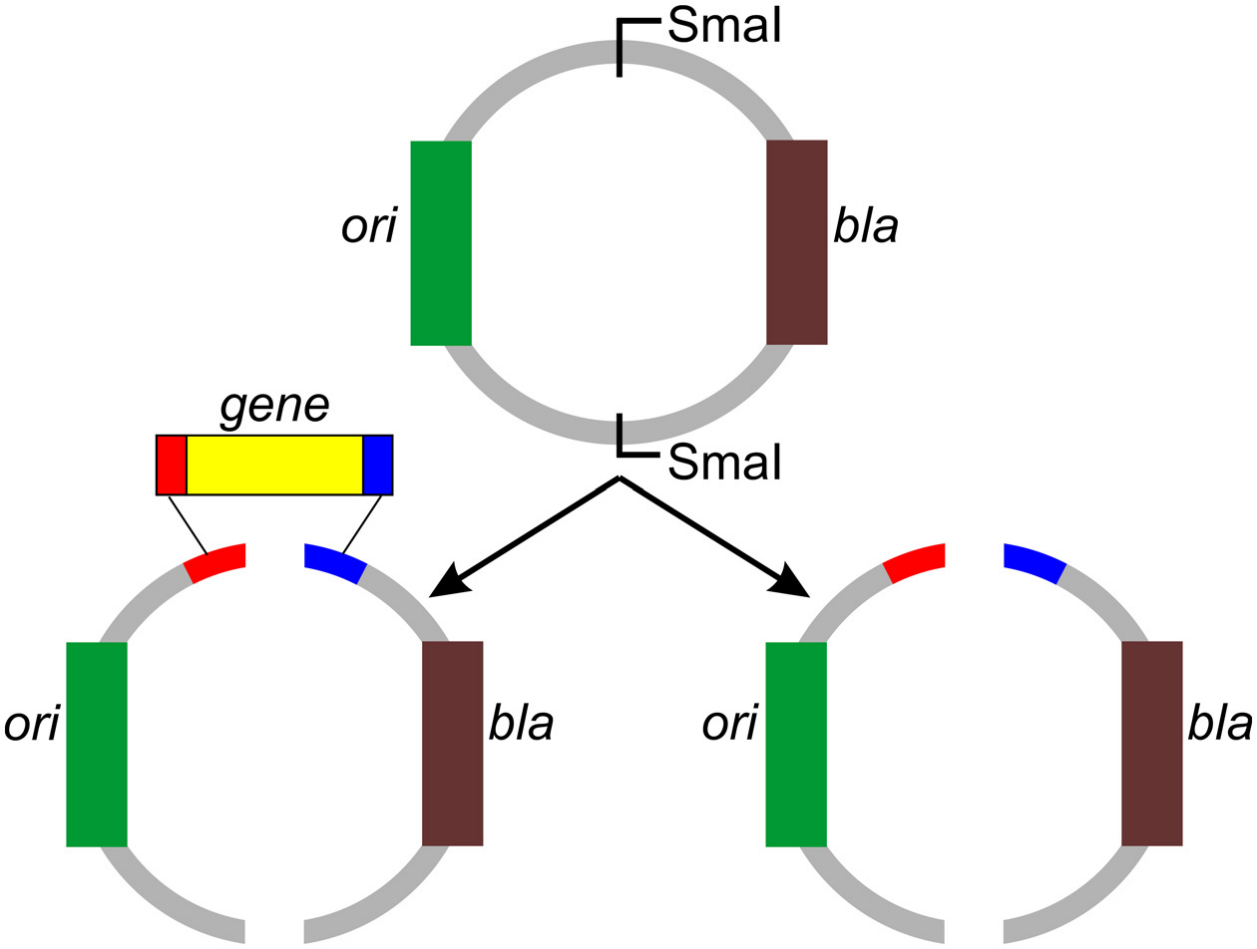
Diagrammatic representation of all-PCR cloning methodology. The vector (pRS-NHS, -CHS, or -NOHS) contains two SmaI sites between *ori* (green box) and *bla* (brown box). Upon digestion with SmaI, two fragments are obtained that are joined together by PCR by means of complementary sequences (shown in red and blue) present both in the vector and in the gene (yellow box). In the control reaction, these fragments will not come together and join since insert is absent.

**Fig 2:**
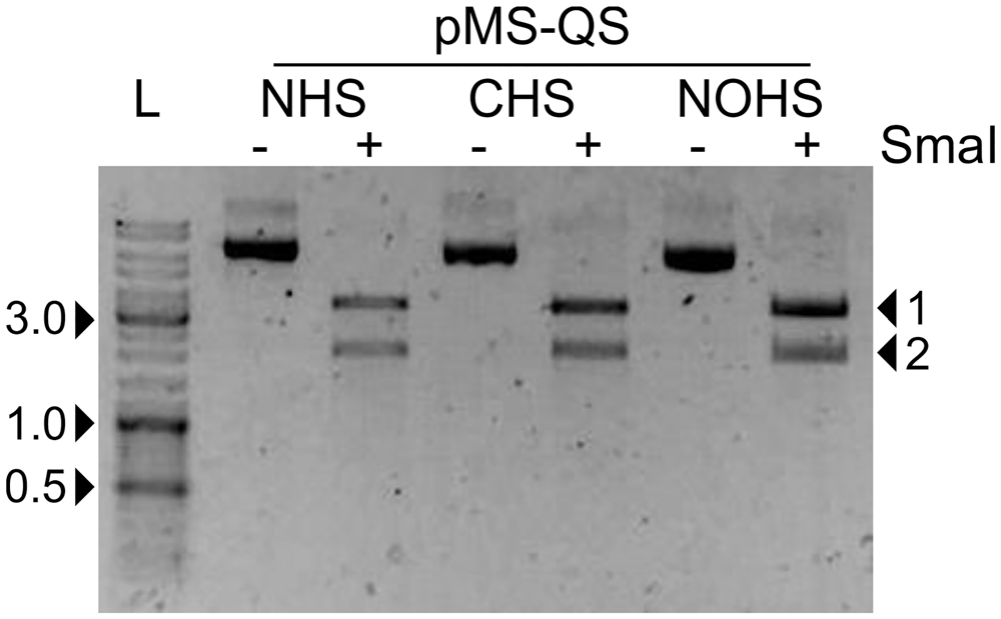
Digestion of pRS vectors yield 2 fragments. The pRS-NHS, pRS-CHS, and pRS-NOHS vectors are digested after inserting an additional SmaI site (refer text). The two fragments thus generated have either *ori* or *bla*. The arrows marked 1 and 2 refer to the vector fragments containing *bla* and *ori*, respectively. ‘-‘ and ‘+’ indicate the absence and presence of SmaI, respectively. ‘L’ refers to DNA ladder.

To isolate the *ori* and the antibiotic-resistance marker gene on separate DNA fragments, we utilized the previously reported Quick-series vectors namely pMS-QS-NHS, pMS-QS-CHS, and pMS-QS-NOHS, which contain a SmaI site for inserting the gene of interest downstream to the T7 promoter [13]. These vectors were first subjected to site-directed mutagenesis (SDM) to introduce another SmaI site between the *ori* and the ampicillin-resistance marker gene (*bla* for β-lactamase) using the ori-Amp-SmaI primer (Table 1). The SDM was carried out essentially as described [17]. The plasmids pRS-NHS, pRS-CHS, and pRS-NOHS thus obtained have the capacity to add an N-terminal 6xHis tag, C-terminal 6xHis-tag, and no histidine tag, respectively, to the expressed protein when cloned at SmaI site originally present in the pMS-QS vectors and also has another SmaI site separating the o*ri* and *bla* gene (Fig 1 and Table 2). These plasmids on further digestion with SmaI yielded the two DNA segments (Fig 2). Here, one of the two fragments contained *ori* while the other fragment retained *bla*. These fragments were subsequently used in overlapping PCR as described below.

**Table 2:** List of plasmids used in this study.

Plasmid	Size (kbp)	Description	Source
pET21b GFP	6.1	*GFP* cloned between NdeI and XhoI sites	AD, IISER Bhopal
pMS-QS-NHS	5.4	Quick-series vector with one SmaI site	Laboratory vectors; Ref. [13]
pMS-QS-CHS	5.3	Quick-series vector with one SmaI site	Laboratory vectors; [13]
pMS-QS-NOHS	5.3	Quick-series vector with one SmaI site	Laboratory vectors; [13]
^1^pRS-NHS	5.4	Rapid series vector with two SmaI sites.	This study
^1^pRS-CHS	5.3	Rapid series vector with two SmaI sites	This study
^1^pRS-NOHS	5.3	Rapid series vector with two SmaI sites	This study
pRS-NHS-GFP	6.1	N-ter His_6_ tagged-GFP	This study
pRS-CHS-GFP	6.0	C-ter His_6_ tagged-GFP	This study
pRS-NOHS-GFP	6.0	GFP without histidine tag	This study
pRS-NHS-adh	6.5	N-ter His_6_ tagged-Adh	This study
pRS-CHS-adh	6.4	C-ter His_6_ tagged-Adh	This study
pRS-NOHS-adh	6.4	Adh without histidine tag	This study
pRS-NHS-rho	6.6	N-ter His_6_ tagged-Rho	This study
pRS-CHS-rho	6.5	C-ter His_6_ tagged-Rho	This study
pRS-NOHS-rho	6.5	Rho without histidine tag	This study

All the plasmids carry ampicillin resistance marker. Histidine-tag, wherever expresses, is mentioned. NHS represents 6x histidine tag at the N-terminus of the expressed protein. Similarly, CHS suggests the presence of His-tag at the C-terminus of the expressed protein. NOHS vectors do not provide the expressed protein with hexa-histidine tag. In all the vectors, the genes are cloned under T7 promoter, as in pET vector. All the plasmids are available upon request.

^1^ The fragments generated after SmaI digestion were used in overlapping PCR reaction.

### Generation of an amplicon of choice

**Fig 3:**
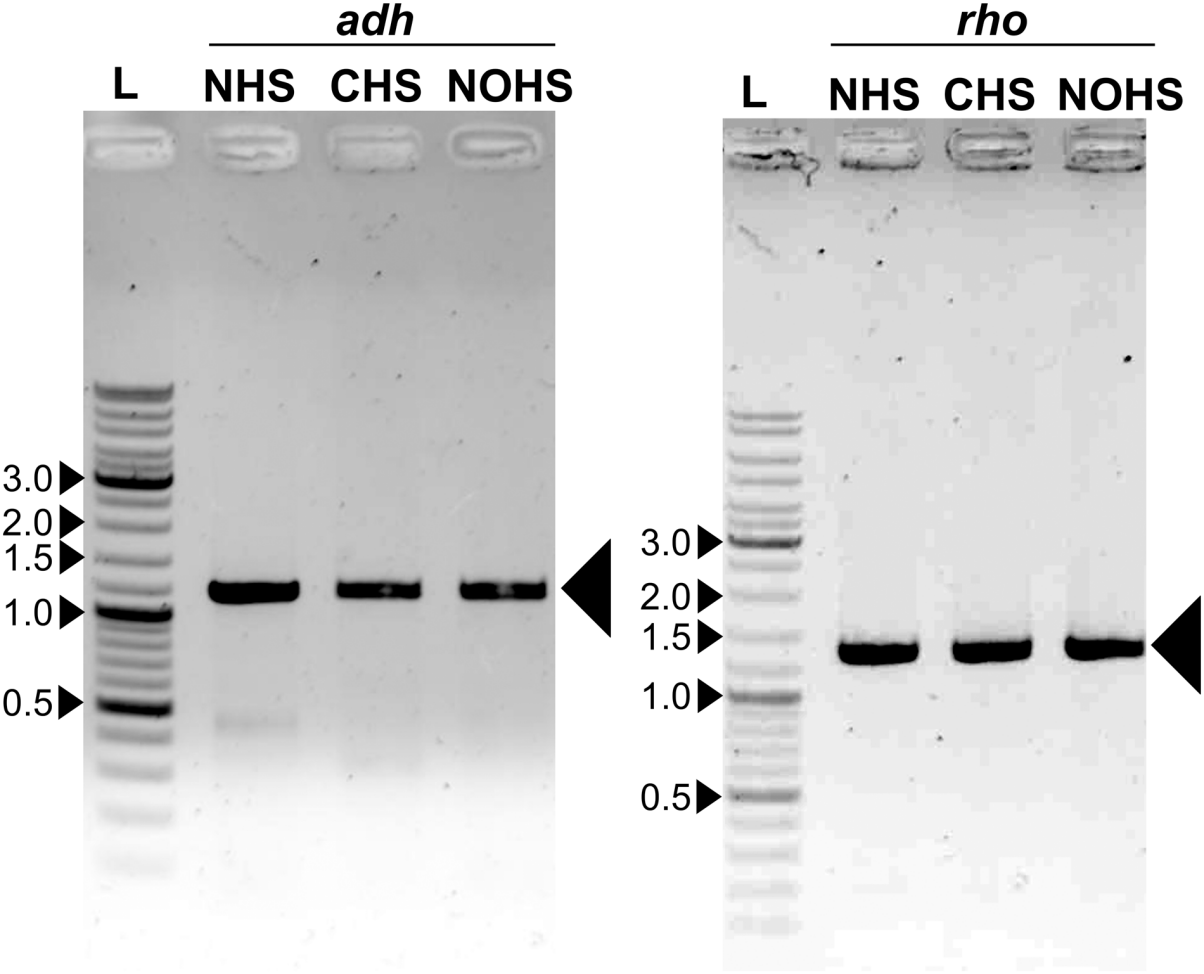
Agarose gel showing the *adh* and *rho* amplicons. Both *adh* and *rho* were PCR amplified using the primers listed in Table 1 and as detailed in the text. Large arrows indicate the amplicon. L represents the DNA ladder; five bands are marked in kbp.

To test our methodology, we first PCR amplified three genes *viz*. *GFP*, *rho*, and *adh* from three different sources for subsequent cloning experiments. The *GFP* (0.7 kbp) was PCR amplified from pET21b vector (Novagen, Madison, WI, USA) that was available in our laboratory (S1 Fig). The *rho* (1.2 kbp) and the *adh* (1.1 kbp) genes were PCR amplified from the genomic DNA of *E*. *coli* BL21(DE3) and *M*. *smegmatis* mc^2^155, respectively (Fig 3) using the primers given in Table 1. All the genes were PCR amplified using different sets of primer pairs to achieve the following: to obtain the N-terminal His-tag in the expressed protein, the gene was amplified using NHS-for and NHS-rev primers and the cloning was carried out in pRS-NHS; similarly, CHS-for and CHS-rev primers were used to amplify and clone the gene in pRS-CHS vector that will yield C-terminal His-tag. To clone the gene in pRS-NOHS, a vector that will not add His-tag, CHS-for and NHS-rev primer combination was used to amplify the gene. The primers were designed such that they carried additional nucleotides that were complementary to the respective vectors (Fig 1 and Table 1).

### A single PCR reaction is required to obtain recombinant clone

**Fig 4:**
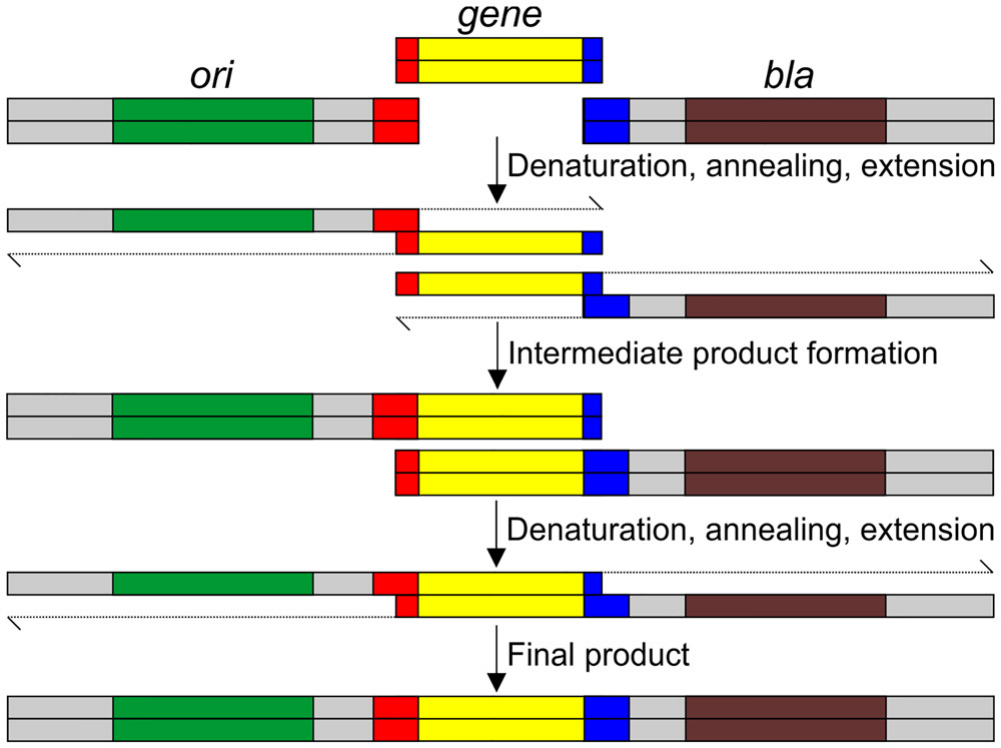
Schematic representation of overlapping PCR methodology. Overlapping PCR method is shown with only the strands that will be extended by the DNA polymerase in PCR; the other strand is omitted for clarity. The extension of the 3’ end by the DNA polymerase is shown. Colour coding is same as in Fig 1. Complementary sequences shown in red and blue will allow the hybridization of the gene with the vector fragments. In the initial PCR cycles, the insert will hybridize with the fragment. The intermediate product formed will contain the insert added to each fragment. Subsequent cycles will allow the extension of the intermediate product to full-length final product, which will be amplified in the remaining cycles with the terminal primers AmpFor and OriFor (not shown).

**Fig 5:**
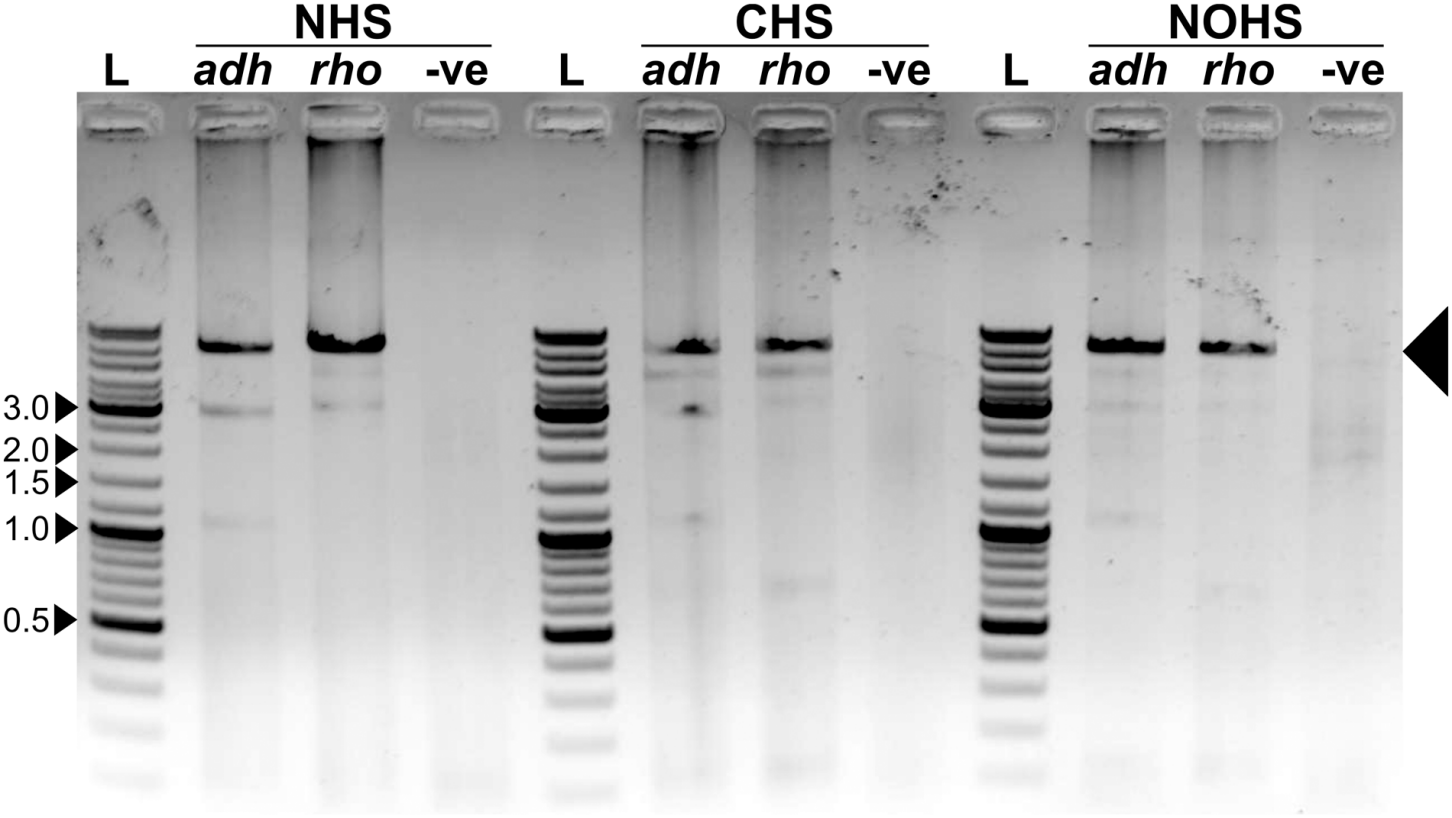
Overlapping PCR result demonstrating the amplification of the full-length vector with the gene inserted. The amplified product is marked with large arrow. L represents the DNA ladder; five bands are marked in kbp. ‘-ve’ represents the negative control that lacked the insert during overlapping PCR reaction. The final product is seen only in those reactions that had the gene of interest. Although other DNA bands are also seen on the gel, they were not identified and, possibly, did not interfere in our experiment.

The final step of our proposed methodology involves an overlapping PCR reaction using the amplicon and the appropriate vector fragments (Fig 4). The PCR reaction was performed with each of the vector fragments, gene amplicon, and the primers—OriFor and AmpFor (Table 1). We performed overlapping PCR for *GFP* insertion using both phosphorylated and non-phosphorylated primers (S2 Fig). Since 5’ phosphate is required in the amplified product to allow ligation of the ends inside the cell after transformation, 5’ phosphorylated primers (purchased from Macrogen, Korea) were used in the overlapping PCR for *adh* and *rho* gene insertion (Fig 5). The annealing temperature of the reaction was set as follows: 64°C for cloning in pRS-NHS, 62°C for pRS-CHS, and 59°C for pRS-NOHS. The overlapping PCR reaction resulted in the formation of complete vector product along with the gene of interest (Fig 5 and S2 Fig). The reaction lacking the gene of interest acted as negative control and showed no amplified product after PCR (Fig 5). This suggests that the gene of interest is required to successfully carry out the overlapping PCR and to produce recombinant DNA.

### DNA constructs generated after overlapping PCR yield all-positive clones

**Fig 6:**
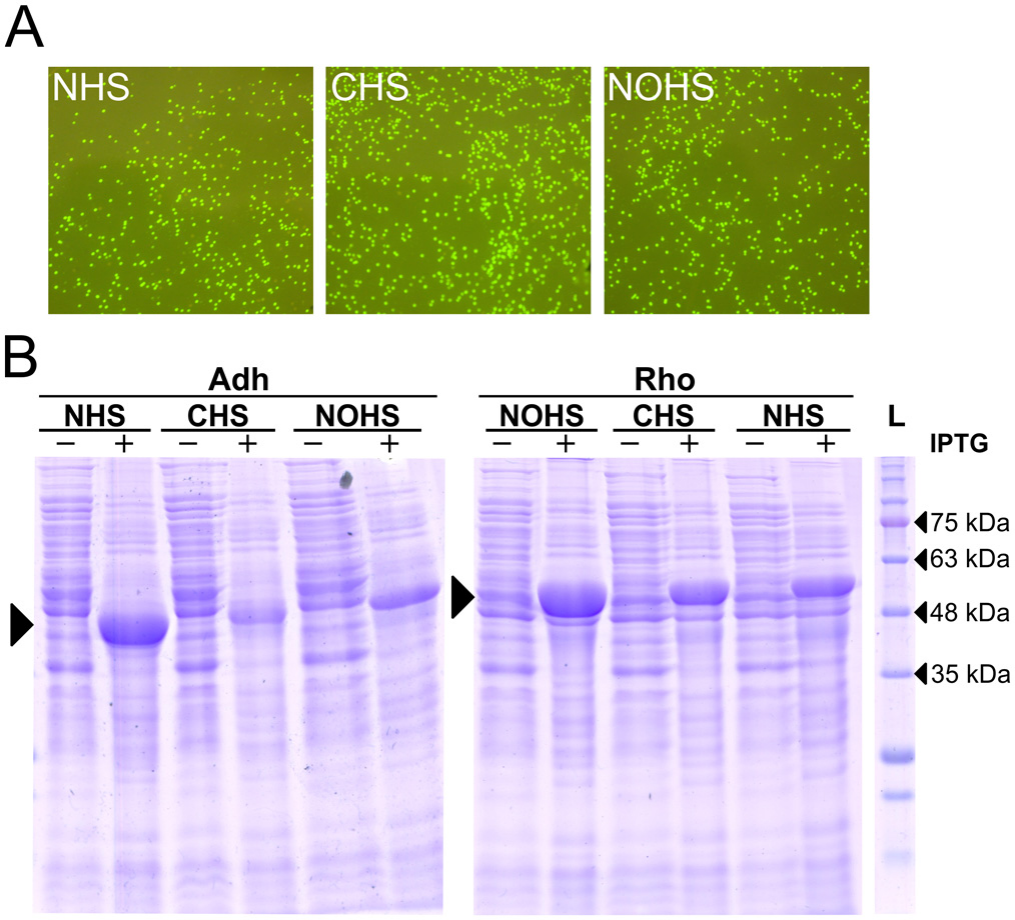
Expression of GFP, Adh and Rho proteins. (A) LB agar plates show the expression of GFP in *E*. *coli* T7 express strain. The plate image was captured in blue light (~400 nm). The overlapping PCR product was purified, ligated, and transformed in *E*. *coli* T7express strain. The agar plate contained the ampicillin and IPTG. A large number of colonies are seen in each case demonstrating the presence of recombinant clones. (B) Induction profile of Adh and Rho proteins in *E*. *coli* BL21(DE3) cells. The large arrows point to the expressed proteins. ‘-‘ and ‘+’ indicate the absence and presence of IPTG, respectively. L represents the protein molecular weight marker; four bands are labelled.

The obtained PCR products were introduced into *E*. *coli* cells as follows. The PCR products generated after overlapping PCR step, were either directly used for transformation or were first subjected to purification using PCR purification kit; the purified product was subsequently used for the transformation. We also performed self-ligation of the PCR product before transformation. After purification, the PCR product was ligated using T4 DNA ligase (Fermentas) and introduced into *E*. *coli* cells. The XL1-blue strain of *E*. *coli* was transformed with PCR products carrying *rho* and *adh* genes while the T7 express strain (NEB) was transformed with *GFP*-containing PCR products. The cells were selected on ampicillin-LB-agar; the medium used for the selection of T7 express additionally contained 1 mM IPTG to directly visualize GFP production. The positive clones for *GFP* cloning were readily identifiable by visualization of green colonies on LB-Amp-IPTG plates under 400nm (blue) light (Fig 6A). We found that the PCR product yield more colonies post PCR clean up and the number of colonies increases many folds after ligating the final product (S3 Fig). A large number of colonies were obtained after transformation with number of recombinant clones reaching up to 100% (Table 3). Here, although the direct transformation of PCR product yields less number of colonies as compared to that observed after self-ligation (S3 Fig and Table 3), we conclude that the ligation of PCR product before transformation is not essential. However, phosphorylated primers are required for these experiments. To confirm the cloning of *rho* and *adh* genes, we performed colony PCR as described previously [13], on at least 16 colonies in each case using pETFor and T7rev primers. Similar to GFP, all the screened colonies were found to be positive (data not shown). Our results thus suggest that the methodology presented here is suitable for rapid cloning of the target gene, and primarily yields positive clones. DNA sequencing data obtained for all the clones further confirmed the integrity of the clones with no mutations in the cloned fragment.

**Table 3:** *GFP* cloning and screening for recombinant clones.

No purification			PCR purification			PCR purification+ligation		
NHS	CHS	NOHS	NHS	CHS	NOHS	NHS	CHS	NOHS
100% (30)	100% (11)	100% (27)	100% (43)	100% (47)	98% (37)	~97% (>200)	~100% (>200)	~100% (>200)

*GFP* was cloned in three vectors using overlapping PCR and the final product was either used directly or after purification, for transformation. The PCR product was also subjected to self-ligation before transformation (PCR purification+ligation). In each case, green fluorescing colonies were counted. Values in parentheses are the number of colonies obtained in each case. The description for NHS, CHS and NOHS is same as given in Table 2.

We next assessed the expression profile of the cloned *rho* and *adh* genes. The recombinant plasmids isolated from randomly picked colonies were introduced in *E*. *coli* BL21(DE3). The protein expression was assessed in all three vectors, i.e. gene with N-terminal, C-terminal, and without 6xHis tag. All the constructs prepared in this study show protein expression (Fig 6B), thus confirming the successful generation of the recombinant plasmids.

## Discussion

PCR is generally the first and the foremost requirement in a molecular cloning experiment. Nevertheless, a complete cloning procedure requires several other steps such as the treatment of the amplicon with restriction enzymes, preparation of the vector backbone, dephosphorylation, ligation, etc. These steps even when performed with all the precautions do not necessarily yield all-positive clones.

With an aim to reduce significantly the time it takes to clone a gene without compromising with the efficiency of getting recombinant clones, we have designed a cloning strategy that involves only two PCR reactions followed by the introduction of the PCR product into the bacteria. Here we have separated the two essential components of a vector DNA—the origin of replication and the antibiotic resistance gene—on two separate DNA fragments. The amplicon of the desired gene obtained from any source (plasmid or genomic DNA) generated in the first PCR reaction is fused with the vector DNA fragments thus bringing the two essential features of the vector on the same DNA molecule. The fusion happens during an overlapping PCR reaction. The fused product yields only recombinant clones since the fragments alone will not help in bacterial survival on an antibiotic-containing agar plate. Furthermore, although in many cases, DpnI treatment is required to remove the parent DNA, this is avoided in our methodology since parent vector fragments will not result in bacterial colony formation. Our all-PCR based cloning methodology reported here allows the desired DNA fragment cloning directly in an expression vector under T7 promoter with a 6x-histidine tag at either terminus, depending upon the chosen vector. Most notably, this protocol is completed in less than 8 hours (for two PCR reactions, amplicon purification, and transformation) and yields only recombinant clones; in these experiments, obtaining a non-recombinant clone, i.e. a vector without the desired insert, is very unlikely. Using GFP, we also show that the amplified DNA can be transformed directly in an expression strain of *E*. *coli* such as T7 express. In addition, we present the cloning and protein expression screening of two genes from different organisms. We are further tempted to add that our methodology of generating recombinant plasmid will help immensely in carrying out cloning in a shuttle vector, where the need to screen for a recombinant clone can be avoided and the final DNA can be introduced immediately in the second host (bacterium, yeast, or higher eukaryotic cell).

The objective of a cloning experiment is to generate recombinant DNA that can be used to synthesize the protein. It is thus highly desirable to develop novel methods that not only ease the cloning process, but also make it extremely rapid in order to save time. We are confident that the methodology presented here will find its use in large scale cloning of the genes for studies in advanced molecular and structural biology.

## Supporting Information

S1 Fig Agarose gel showing the *GFP* amplicons. The *GFP* gene was PCR amplified using the primers listed in Table 1 and as detailed in the text. L represents the DNA ladder; two bands are marked in kbp.
(TIF)

S2 Fig Overlapping PCR result for *GFP* gene insertion in the vector. The amplified product is marked with large arrow. L represents the DNA ladder; three bands are marked in kbp. ‘P’ and ‘DeP’ refer to reaction containing phosphorylated and dephosphorylated primers (AmpFor and OriFor; refer Table 1), respectively. Although other DNA bands are also seen on the gel, they were not identified and, possibly, did not interfere in our experiment.
(TIF)

S3 Fig Agar plate image showing the expression of GFP in *E*. *coli* T7 express strain. The overlapping PCR product was transformed in *E*. *coli* T7 express strain after with and without ligation and purification. The plates were imaged using blue light and SYBR Gold Filter (485–655 nm) in a gel documentation system (UVP, LLC). The agar plate contained the ampicillin and IPTG. A large number of colonies are seen in the case of ligation after purification of the PCR product. Since direct transformation of PCR product also yields recombinant colonies, post-processing of the PCR reaction mixture is not required.
(TIF)

## Abbreviations

PCR: polymerase chain reaction
IPTG: Isopropyl β-D-thiogalactopyranoside
PAGE: polyacrylamide gel electrophoresis
kbp: kilo basepair
SDM: site directed mutagenesis
