# Supplementary figures and images for: Rapid and Robust PCR-Based All-Recombinant Cloning Methodology
Source: PLoS One. 2016 Mar 23;11(3):e0152106. doi: 10.1371/journal.pone.0152106 (PMC4805250; doi:10.1371/journal.pone.0152106)

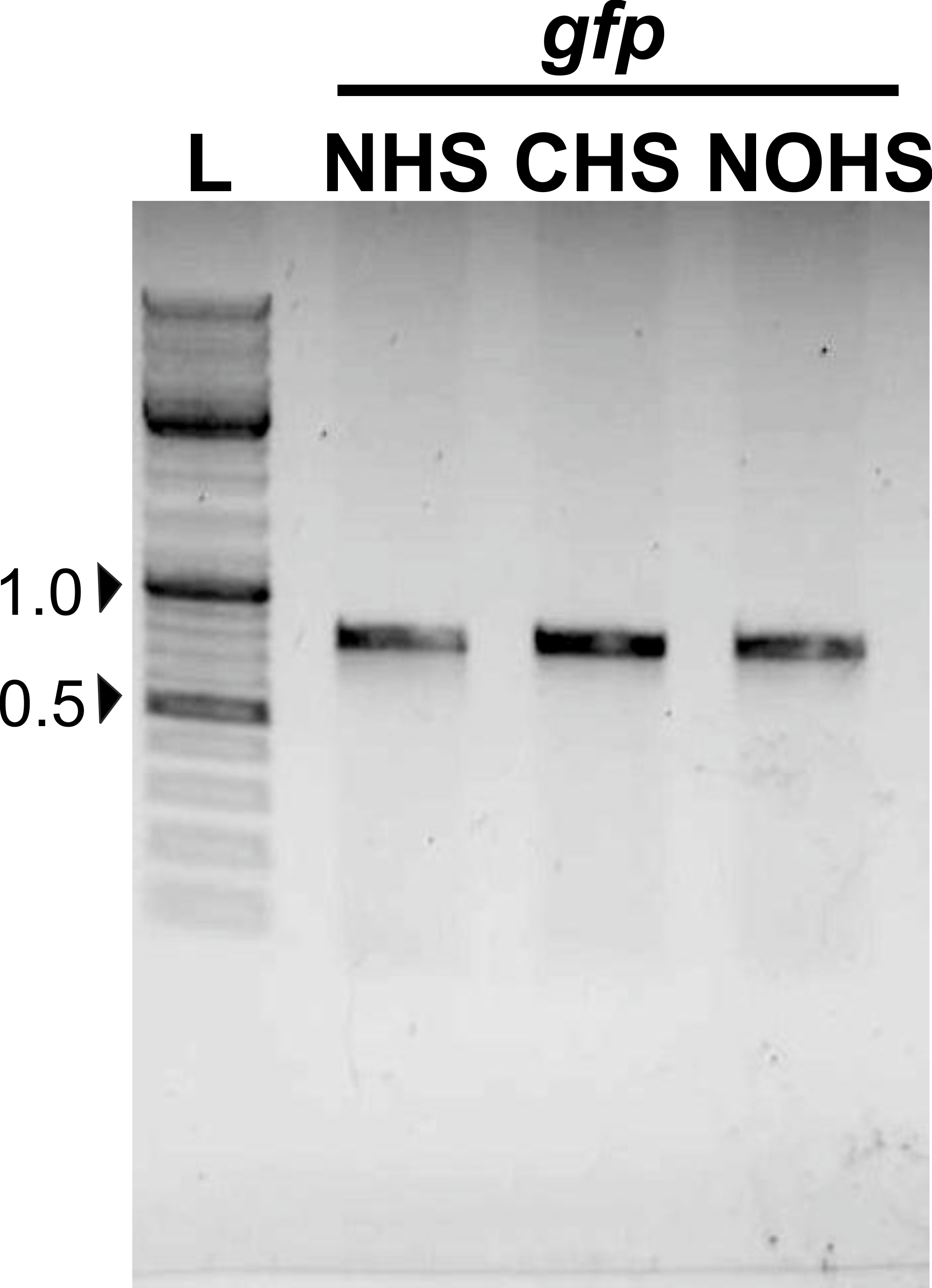

Supplement: S1 Fig — The GFP gene was PCR amplified using the primers listed in Table 1 and as detailed in the text. L represents the DNA ladder; two bands are marked in kbp. (TIF) [file pone.0152106.s001.tif]

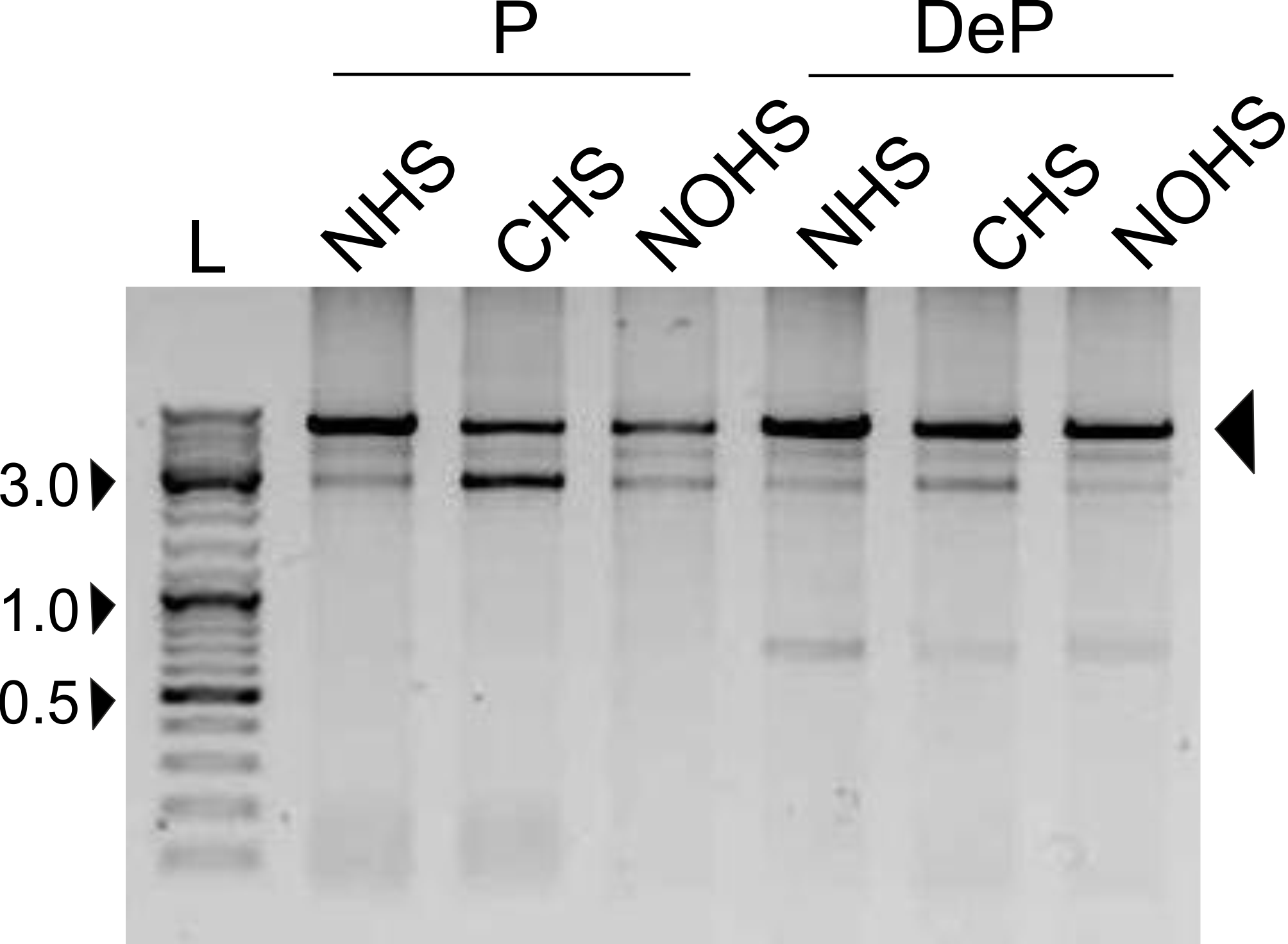

Supplement: S2 Fig — The amplified product is marked with large arrow. L represents the DNA ladder; three bands are marked in kbp. ‘P’ and ‘DeP’ refer to reaction containing phosphorylated and dephosphorylated primers (AmpFor and OriFor; refer Table 1), respectively. Although other DNA bands are also seen on the gel, they were not identified and, possibly, did not interfere in our experiment. (TIF) [file pone.0152106.s002.tif]

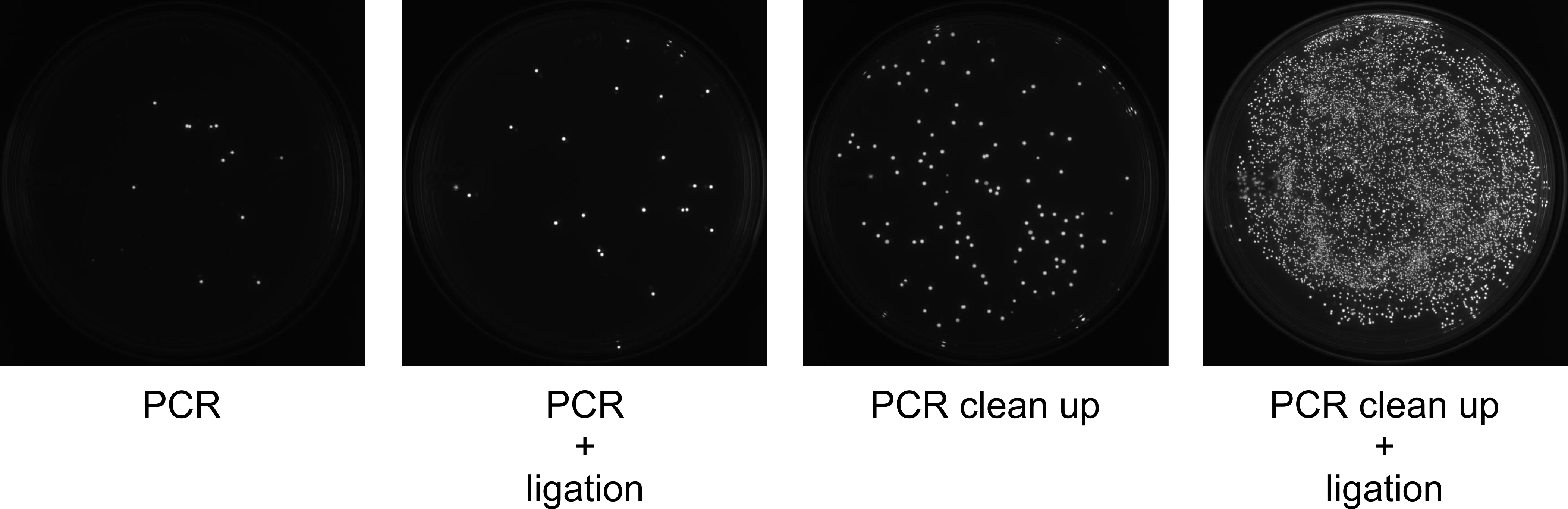

Supplement: S3 Fig — The overlapping PCR product was transformed in E. coli T7 express strain after with and without ligation and purification. The plates were imaged using blue light and SYBR Gold Filter (485–655 nm) in a gel documentation system (UVP, LLC). The agar plate contained the ampicillin and IPTG. A large number of colonies are seen in the case of ligation after purification of the PCR product. Since direct transformation of PCR product also yields recombinant colonies, post-processing of the PCR reaction mixture is not required. (TIF) [file pone.0152106.s003.tif]
